# Genome-Wide SNP Signal Intensity Scanning Revealed Genes Differentiating Cows with Ovarian Pathologies from Healthy Cows

**DOI:** 10.3390/s17081920

**Published:** 2017-08-21

**Authors:** Ricardo Salomón-Torres, Martin F. Montaño-Gómez, Rafael Villa-Angulo, Víctor M. González-Vizcarra, Carlos Villa-Angulo, Gerardo E. Medina-Basulto, Noé Ortiz-Uribe, Padmanabhan Mahadevan, Víctor H. Yaurima-Basaldúa

**Affiliations:** 1 Departamento de Posgrado, Universidad Estatal de Sonora, S.L.R.C., Sonora 83500, Mexico; sinhuet7@hotmail.com (N.O.-U.); vyaurima@yahoo.com (V.H.Y.-B.); 2 Instituto de Investigaciones en Ciencias Veterinarias, Universidad Autónoma de Baja California, Baja California 21386, Mexico; martinmg@uabc.edu.mx (M.F.M.-G.); vvizcarra@uabc.edu.mx (V.M.G.-V.); gerardom@uabc.edu.mx (G.E.M.-B.); 3 Laboratorio de Bioinformática y Biofotonica, Instituto de Ingeniería, Universidad Autónoma de Baja California, Baja California 21100, Mexico; villac@uabc.edu.mx; 4 Department of Biology, University of Tampa, Tampa, FL 33606, USA; pmahadevan1@gmail.com

**Keywords:** SNP, ovarian cysts, Holstein cattle, Axiom Genome-Wide Bos 1 array, bioinformatics

## Abstract

Hypoplasia and ovarian cysts are the most common ovarian pathologies in cattle. In this genome-wide study we analyzed the signal intensity of 648,315 Single Nucleotide Polymorphisms (SNPs) and identified 1338 genes differentiating cows with ovarian pathologies from healthy cows. The sample consisted of six cows presenting an ovarian pathology and six healthy cows. SNP signal intensities were measured with a genotyping process using the Axiom Genome-Wide BOS 1 SNPchip. Statistical tests for equality of variance and mean were applied to SNP intensities, and significance *p*-values were obtained. A Benjamini-Hochberg multiple testing correction reveled significant SNPs. Corresponding genes were identified using the Bovine Genome UMD 3.1 annotation. Principal Components Analysis (PCA) confirmed differentiation. An analysis of Copy Number Variations (CNVs), obtained from signal intensities, revealed no evidence of association between ovarian pathologies and CNVs. In addition, a haplotype frequency analysis showed no association with ovarian pathologies. Results show that SNP signal intensity, which captures not only information for base-pair genotypes elucidation, but the amount of fluorescence nucleotide synthetization produced in an enzymatic reaction, is a rich source of information that, by itself or in combination with base-pair genotypes, might be used to implement differentiation, prediction and diagnostic procedures, increasing the scope of applications for Genotyping Microarrays.

## 1. Introduction

Hypoplasia and ovarian cysts are two of the most common pathologies in cattle. Ovarian hypoplasia is a congenital malformation that is characterized by the lack of proper growth and development of the ovaries, presenting a decreased or subnormal size. The literature has reported that ovarian hypoplasia can occur in one or both ovaries. However, when it occurs in one ovary of the animal (unilateral hypoplasia), the remaining one is also affected. Only 9% of females suffer from a bilateral hypoplasia together with an offspring’s womb and an offspring’s mammary gland [1]. On the other hand, ovarian cysts can be found in one or both ovaries, and are dynamic structures described as multiple or single anovulatory follicles that persist for 7 days in the presence of low progesterone concentrations. Ovarian cysts have a diameter greater than 18 mm, absence of a corpus luteum, and a lack of uterine tonicity [2]. Generally, it is accepted that this pathology is caused by a dysfunction of the hypothalamic-pituitary-gonadal axis due to endogenous or exogenous factors [3,4,5]. In dairy cattle, ovarian cysts commonly appear in the postpartum period and are mainly responsible for reproductive problems due to a prolongation generated in calving to first estrus interval, as well as calving to conception and birth-delivery. Ovarian cysts have a low incidence in beef cattle and are more common in dual purpose breeds [6]. However, in dairy cattle it typically ranges between 5% and 10%, and may extend up to 30%, depending on the country, the facilities and the animals’ management [7,8,9]. Nutrition, uterine infection, stress, milk production, age, reproductive time, body condition and the season, combined with inherited genetics are all factors that contribute to the development of ovarian cysts [6].

Single Nucleotide Polymorphism (SNP) Genotyping microarray biotechnology is currently widely used for genome-wide studies, due to its capability of simultaneously measuring the allelic content from a specific position in millions of DNA fragments [10,11]. By sensing the intensity of fluorescence produced by an enzymatic nucleotide synthesis reaction, it is possible to elucidate base-pair genotypes across polymorphic positions genome-wide. Most practical applications have been focused on the use of signal intensities just to elucidate the allelic content of SNP genotypes, and then use the genotypes to estimate correlations and association with phenotypes [12]. Just a few practical applications have focused on the use of SNP signal intensities to directly associate SNP position with phenotypes, from which the discovery of copy number variations (CNVs) and neutral loss of heterozygosity (LOH) are the most widely used [13].

In this work we present a new application of SNP signal intensities. We performed a genome-wide SNP signal intensity scanning looking for genes differentiating between cows suffering from ovarian pathologies and healthy cows. The data consisted of 648,315 informative SNP markers assayed with the Axiom Genome-Wide Array Bos 1 (from Affymetrix Inc., Santa Clara, CA, USA) on 12 cows, from which six were suffering ovarian pathologies. We present a methodology based on the normalized signal intensity that is capable of distinguishing between the two sets of data (disease and healthy cows). The methodology implements a genome-wide scanning, using statistical tests for equality of variance and mean, to vectors of normalized signal intensities, and a PCA clustering analysis for differentiation. Examination of *p-*values and cluster visualization allowed us to establish differences between data at the signal intensity level that was not possible at the haplotype level.

## 2. Material and Methods

### 2.1. Ethical Issues

The Autonomous University of Baja California (UABC, Baja California, Mexico) animal care and use committee deemed it unnecessary to obtain ethical clearance for this study, as all blood samples used for DNA extraction were collected under the directives on animal research of the Institute for Research in Veterinary Science UABC (IICV-UABC) on the basis of the Mexican laws on animal studies (NOM-003-ZOO-1994 and NOM-062-ZOO-1999).

### 2.2. Animal Samples and DNA Extraction

None of the animal samples were related to each other. They came from the experimental Holstein cattle herd of the Veterinary Science Research Institute of the Autonomous University of Baja California, in Mexicali (Baja California, Mexico). The samples consisted of blood, obtained by venipuncture of the coccygeal vein, using vacutainer tubes (Vacutainer, Hemogar^®^, Franklin Lakes, NJ, USA) with EDTA anticoagulant. DNA extraction and purification was performed using a QIAGEN kit (QIAamp DNA Blood, catalogues 51104 and 51106; QIAGEN^®^, Hilden, Germany). All DNA samples were analyzed by spectroscopy and agarose gel electrophoresis, and stored at −20 °C for future analysis.

### 2.3. Post-Mortem Detection of Ovarian Pathologies and Genotyping

Due to the reduction in their reproductive and productive capacity, a group of cows were slaughtered following the official Mexican standard for humane treatment in the slaughter of comestible animals (NOM-008-ZOO-994 and NOM-033-ZOO-1995). In order to find a possible cause to justify their null reproduction and low milk production, a physical examination was performed following the procedures defined by Sisson et al. [14]. In the inspection of their reproductive systems, we found that six animals exhibited pathologies associated with their ovaries. Once animals that suffered an ovarian pathology were identified, their DNA was recovered from frozen conservation. In addition, genome-wide genotyping was performed, adding DNA from six healthy cows. Supplementary File 1: Table S1 contains a description of the cows’ data. The 12 DNA samples were genotyped with the Axiom Genome-Wide BOS 1 Array with an average call rate for each individual sample of 99.5%.

### 2.4. Signal Intensity Normalization

The signal intensity was generated from raw data (CEL files), following the procedure defined by Wang et al. [15]. We obtained two signal intensity values for each SNP in each sample. Then signal intensities were normalized by expressing them as Log**_2_** ratio. The calculation was made using a Perl script with the following procedure: first, for each marker, a reference was developed using the equation T = A + B, where A and B are the signal intensity values of each allele. For each SNP, a reference is established using M = median (T_sample1, T_sample2, …, T_sampleN). The second step is the estimation of the intensity for each individual sample using the equation log**_2_** (T/M) [16].

### 2.5. Gene Selection for the Study

The complete list of genes in the autosomal chromosomes (approx. 35,000) were obtained from the bovine genome *Bos taurus* (cattle) genome viewer (release annotation 104) from NCBI Map Viewer (http://www.ncbi.nlm.nih.gov/mapview/). From this list, based on the criteria for SNP number selection for defining Structural Variations used by Salomon-Torres et al. [11], we selected the genes that had at least 15 SNPs within their DNA sequence with the purpose of increasing accuracy. As a result, 4532 genes were selected for the analysis of their signal intensity. Regarding the identified hormone genes involved in ovarian pathology processes, at least 11 were identified as highly related to ovarian diseases (see Supplementary File 1: Table S2, for a complete list). Only two genes met the criteria (≥15 SNPs): Follicle stimulating hormone receptor (*FSHR*) and the Luteinizing hormone/choriogonadotropin receptor (*LHCGR*), with 61 and 19 SNPs respectively.

### 2.6. Identification of Copy Number Variations

We performed CNVs prediction in our dataset using the PennCNV and QuantiSNP algorithms [15,17]. PennCNV was executed using the -*test* option using default values. QuantiSNP algorithm was executed enabling the options-isaffy and -levels, since we used an Affymetrix array. We considered at least three adjacent SNPs indicating a loss or gain, with a full length greater or equal to 1 kb, to declare a putative CNV as identified by any of these two algorithms. In addition, we compared results with previously reported CNVs [12,18,19,20,21,22,23,24].

### 2.7. Principal Components Analysis

Formally, PCA is defined as an orthogonal linear transformation that transforms the data to a new coordinate system such that the greatest variance by any projection of the data comes to lie on the first coordinate (called the first principal component), the second greatest variance on the second coordinate, and so on. PCA is theoretically the optimum transform for a given data in least square terms. The procedure for obtaining PCAs can be summarized as follows:

Given a vector **X^T^** of *n* dimensions, *X^T^* = [*x*_1_, *x*_2_, *…* , *x_n_*]*^T^*, whose mean vector **M** and covariance **C** are described by:

*M = E* (*X*) = [*m***_1_**, *m***_2_**, … *m***_n_**]**^T^**

*C* = *E* [(*X* − *M*) (*X* − *M*)***^T^***]

Calculate the eigenvalues λ_1_, λ_2_, …, λ_3_, and the eigenvectors *P*_1_, *P*_2_, …, *P_d_*; arrange them according to their magnitude.

λ_1_ ≥ λ_2_ ≥ … λ**_n_**

Select *d* eigenvectors to represent the *n* variables, *d* < *n*. Then, the *P*_1_, *P*_2_, …, *P_d_* are called the principal components [25]. We used R software to perform this analysis [26].

### 2.8. Haplotype Inference and Haplotype Blocks

We estimated the haplotype pair for each sample using PHASE 2.1 algorithm [27,28]. The program PHASE implements a Bayesian statistical method for reconstructing haplotypes from population genotype data. For identification of haplotype blocks, we used HAPLOVIEW 4.2 algorithm [29]. Haploview calculates several pairwise measures of LD, which it uses to create a graphical representation and the user has the option to select one of several commonly used block definitions to partition the region into segments of strong LD.

### 2.9. Correction for Multiple Testing

A Benjamini and Hochberg correction for multiple testing was applied to *p*-values in order to control the False Discovery Rate (FDR). The approach was implemented as follows: first, all *p*-values were ordered from smallest to largest denoting the *i*-th smallest *p*-value by *P(i)*, for each *i* between 1 and *m* (*m* is the total number of *p*-values), then, starting from the largest *p*-value *P(m)*, compare *P(m)* with 0.05 x *i/m*. Continue as long as *P(i)* > 0.05 x *i/m.* Let *k* be the first time when *P(k)* is less than or equal to 0.05 x *k/m*, and declare the differences corresponding to the smallest *k p*-values as significant [30].

## 3. Results

### 3.1. Differentiation between Disease and Healthy Samples

We inspected the complete list of autosomal genes (~35,000 genes) and selected those having at least 15 SNPs within their DNA sequence. As a result, 4532 genes were selected for this study. For each selected gene, two vectors were generated. The first vector included the normalized signal intensity from the six animals suffering the disease. The second vector included the normalized signal intensity from the healthy animals. An *F-*test for equality of variance and a *T*-test for equality of means were applied to each of the 4532 selected genes. After applying Benjamini-Hochberg multiple testing correction, only the *p-*values of 1338 genes resulted significant.

From the 1338 significant genes, 729 are the best reference sequences (RefSeq best), 414 are messenger RNA (mRNA), 162 are uncharacterized RNA sequences (maseq), and 33 code for proteins. The chromosomes with the highest number of involved genes were chromosome four, one and two, with 88, 82 and 83 genes, respectively. Chromosomes with the lowest number of involved genes were twenty nine, twenty eight and seven, with 17, 20, and 21 genes respectively. The family groups with more genes involved were zinc finger with 28 genes, solute carrier family with 17, and family with similar sequence with 15 genes. The gene with the greatest number of SNPs within its sequence was *RBFOX1* (RNA binding protein fox-1 *C. elegans* homolog 1) with 970, and had the smallest *p-*value of 1.57 × 10^−16^. In addition 55 genes had the lowest number of SNPs within their sequence, with 15. For more details, see Supplementary File 1: Table S3.

As a measure of accuracy, we inspected the results of the statistical analysis of two genes that are well documented as highly related to ovarian diseases: the gene *FSHR*, which is a follicle stimulating-hormone, and the gene *LHCGR*, which is a luteinizing/choriogonadotropin receptor hormone [31,32,33]. We also examined five groups of SNPs placed around the two genes (out of the gene), and the gene *LOC537580* known to have no relation to ovarian disease. We obtained a *p-*value of 0.0002 for the *FSHR* gene, indicating a highly significant difference between disease and healthy samples. In contrast, an average *p*-value of 0.44 was obtained for a group of five SNPs to the right, and a group of five SNPs to the left of the *FSHR* gene location, indicating no difference between disease and healthy samples. Consistently, for the *LHCGR* gene, we obtained a *p-*value of 0.0013, while we obtained an average *p-*value of 0.33 for the surrounding SNP groups. Additionally, we randomly selected a DNA region without encoded genes and with no relationship to ovarian functions in cows. The region start position is at 32,108,743 and an ending position at 32,305,585 in chromosome 20. The total length is 196,842 base-pairs covering 40 SNPs. A *p-*value of 0.58 was obtained for this region, showing no difference in the mean signal intensity between the two groups.

In order to investigate if interaction exists between genes found significant in this study, we did a search in the KEGG Pathway Database (http://www.genome.jp/kegg/pathway.html) [34] for molecular interactions and reaction networks. We found 54 genes involved in ovarian steroidogenesis (bta04913), from which 21 contain at least 15 SNPs. In addition, we found that *FSHR* and *LHCGR* genes are involved in the Calcium Signaling Pathway (bta04020), in the cAMP Signaling Pathway (bta04024) for signal transduction, in the neuroactive ligand-receptor interaction (bta04080) for signaling molecules and interaction in the environmental information processing section, and in the prolactin signaling pathway from endocrine system (see Table 1).

### 3.2. Application of PCA for Differentiation

**Figure 1:**
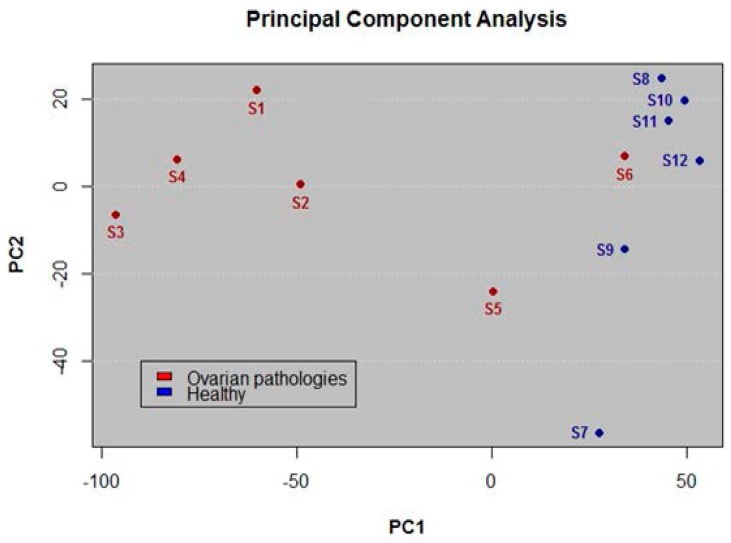
PCA of 1338 significant genes. Plot of the PC1 versus PC2. Clear differentiation is observed between the two study groups.

In addition to the previous analysis, we conducted a PCA using the initial vectors generated for each animal and each gene. The results show clear differentiation between the two kinds of samples. Figure 1 shows PC1 vs PC2 including the 1338 significant genes. Supplementary File 1: Table S4 contains the data for calculating PCA. We can see that the dispersion of samples that belong to the group of cows suffering an ovarian pathology (red dots in the plot) appear separated from the samples that belong to the group of healthy cows (blue dots in the plot). Healthy cows had negative loadings (except S6 which appears with positive loading), while sick cows had positive loadings.

**Figure 2:**
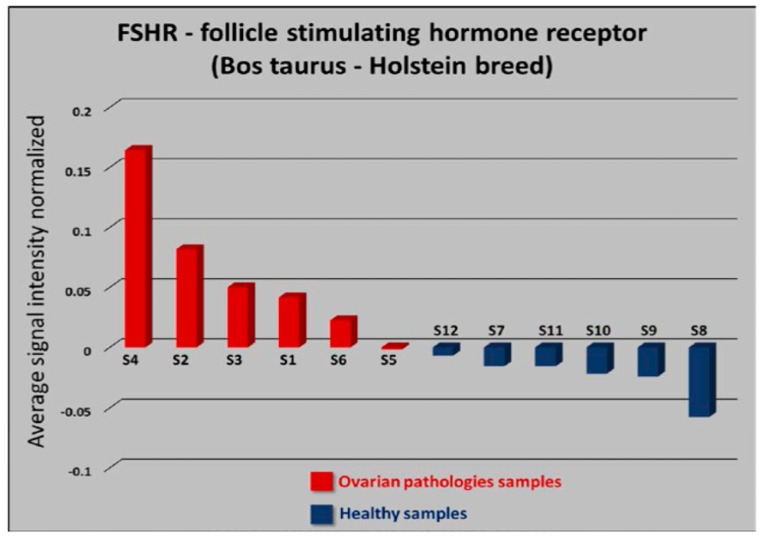
Principal component analysis for Follicle stimulating hormone receptor. The plot shows PC1 sorted average loading values of normalized signal intensity for every sample.

The differentiation appears even clearer when we apply PCA individually to genes we know are strongly related to ovarian pathologies. Figure 2 shows a bar plot of normalized values of PC1 loadings for the gene *FSHR*. In the plot, bars represent average loading values of normalized signal intensity for every sample. 

### 3.3. Searching for Association between CNVs and COFs

In order to identify a possible association between copy number variants (CNVs) and cystic ovarian follicles (COFs), we performed a CNV detection using the signal intensities from the six animals with ovarian pathology. We used two different algorithms, PennCNV and QuantiSNP, with default parameters, and found 84 and 111 CNVs, respectively. None of them showed overlapping with the hormone genes involved in ovarian pathology processes considered in this study. In addition, we performed a review of several studies reporting CNVs, from a total of 6580 samples from different cattle breeds [12,18,19,20,21,22,23,24]. We found just one CNV reported by Bickhart et al. [19] falling in the *FSHR* gene from this study.

### 3.4. Searching for Association between Haplotypes and COFs

**Figure 3:**
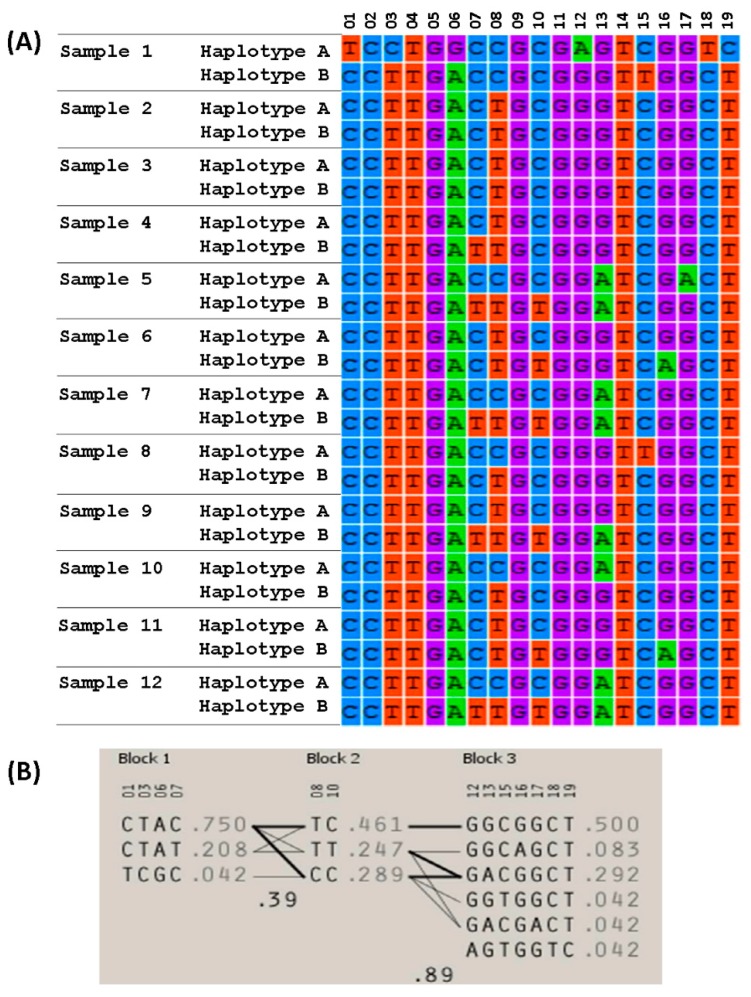
Inferred haplotypes for *LHCGR* gene. Panel (**A**) shows the 12 samples analyzed and their two inferred haplotypes, panel (**B**) shows the haplotype blocks and their frequencies.

In order to discover if there were haplotypes associated to COFs, we obtained the base-pair allele genotypes for all SNPs covered by the genes in the study. We used the files AxiomGT1.Calls.TXT and Axiom_GW_Bos_SNP_1.na32.annot.CSV (provided by the Affymetrix genotyping service) which contain the final cluster of genotypes and annotation for cattle UMD3.1 annotation. For each gene, we inferred haplotype pairs using PHASE 2.1 algorithm [27,28], and defined haplotype blocks using the Haploview 4.2 software [29]. We did not find any haplotype block, or haplotype pattern, consistent just for one group, either healthy or sick. Figure 3 shows the haplotype pairs from the 12 samples, and for all the 19 SNPs covered by the *LHCGR* gene. In this example, as in the rest, we found no evidence of haplotype association.

## 4. Discussion

In principle, all genes from the bovine genome were candidates to perform the differentiation study, but due to the selection criteria, only 1338 were declared as significant genes. From these genes, *FSHR* and *LHCGR* were selected for further analysis because it is known they are highly related to ovarian diseases. For differentiation between disease and healthy groups, the normalization of SNP signal intensities allowed us to implement statistical tests for equality of variance and mean, which in turn gave us the first insight of differentiation, based on a measure of data location. From the four differentiation tests (*FSHR* and *LHCGR* associated genes, *LOC537580* non associated gene, and a random region), all confirmed the same results. The first two with a clear differentiation and the other two with no differentiation at all, judging by *p-*values from statistical test. As a complementary technique, PCA allowed us to make differentiation based on data variability. The results presented in (Figure 1) show the dispersion of samples in a two dimensional plot. Sample S6, which belongs to disease group, is located close to healthy group, but it does not mix in with them. 

From the CNVs analysis, because none of the CNVs detected in this study were related to the hormones involved in ovarian pathology processes described in this study, we suggest that COFs and the other ovarian pathologies do not originate in CNVs. The comparison made with previous studies reporting CNVs and other structural variations (considering healthy animals) was to verify if the sequences of the genes from the hormones in our study were affected by the recombination process and also if an aberration generates the formation of CNVs. Our results showed that from the 6736 CNVRs previously reported in the literature, only 4.45 × 10^−**4**^% overlap with the hormones of our study. Hence, we assume that the formation of CNVs in these regions might be due to other factors not directly related to problems in the ovaries.

The fact that there were no haplotype blocks, or haplotype patterns, consistent just for one group, either healthy or disease, makes clear that this differentiation analysis is not possible at the genotype, or haplotype level. This also shows the missing of important information when mapping from signal intensities to base-pair genotypes information. Therefore, complementing information at the intensity level, with information at the genotype level might increase the power of analysis and the scope of applications for SNP genotyping microarrays.

## 5. Conclusions

This work presents the first genome-wide SNP signal intensity scanning looking for genes differentiating cows suffering ovarian pathologies from healthy cows. Statistical test for equality of mean and PCA of signal intensity provided by a SNP genotyping microarray revealed a total of 1338 differentiating genes. Differentiation achieved from signal intensity was not possible at the genotype, or haplotype level, which highlighted the missing of important information when mapping from signal intensities to base-pair genotypes information.

Finally, our results show that SNP signal intensity, which captures not just information for base-pair genotypes elucidation, but the amount of fluorescence nucleotides synthetization produced in an enzymatic reaction, is a rich source of information that, by itself, or in combination with base-pair genotypes, might be used to implement differentiation, prediction, and diagnostic procedures, increasing the scope of applications for genotyping microarrays.

### Data Access

The complete raw data, supporting the results are available in the ArrayExpress repository, under the accession number E-MTAB-4874 in http://www.ebi.ac.uk/arrayexpress.

## Figures and Tables

**Table 1:** Molecular interactions and reaction networks from the KEGG Pathway Database where genes *FSHR* and *LHCGR* are involved. Entry column corresponds to the id. Name column is the process to which it belongs. KEGG genes column is the number of genes that interact in the network. Total genes column is the number of genes that had at least 15 SNPs. Coverage genes column is the percentage of genes found in KEGG. Non-significant genes column is the number of genes that failed the Benjamini-Hochberg adjustment test. The Significant genes column is the number of genes that passed the test.

Entry	Name	KEGG Genes	Total Genes	Coverage Genes	Non-Significant Genes	Significant Genes
bta04020	Calcium signaling pathway	189	62	32.8%	39	23
bta04024	cAMP signaling pathway	199	59	29.64%	39	20
bta04080	Neuroactive ligand-receptor interaction	303	61	20.13%	46	15
bta04913	Ovarian steroidogenesis	52	19	36.53%	9	10
bta04917	Prolactin signaling pathway	76	19	25%	12	7

## Supplementary Materials

The following are available online at http://www.mdpi.com/1424-8220/17/8/1920/s1, Supplementary File 1: Table S1: Description of the sample, Table S2: Hormones genes and SNPS using for this study, Table S3: Results of the *T*-test applied to 4532 genes, Table S4: Data for calculate PCA.
